# Evidence of Alternative Cystatin C Signal Sequence Cleavage Which Is Influenced by the A25T Polymorphism

**DOI:** 10.1371/journal.pone.0147684

**Published:** 2016-02-04

**Authors:** Annie Nguyen, John D. Hulleman

**Affiliations:** 1 Department of Ophthalmology, University of Texas Southwestern Medical Center, Dallas, TX, United States of America; 2 Department of Pharmacology, University of Texas Southwestern Medical Center, Dallas, TX, United States of America; Sungkyunkwan University, REPUBLIC OF KOREA

## Abstract

Cystatin C (Cys C) is a small, potent, cysteine protease inhibitor. An Ala25Thr (A25T) polymorphism in Cys C has been associated with both macular degeneration and late-onset Alzheimer’s disease. Previously, studies have suggested that this polymorphism may compromise the secretion of Cys C. Interestingly, we found that untagged A25T, A25T tagged C-terminally with FLAG, or A25T FLAG followed by green fluorescent protein (GFP), were all secreted as efficiently from immortalized human cells as their wild-type (WT) counterparts (e.g., 112%, 100%, and 88% of WT levels from HEK-293T cells, respectively). Supporting these observations, WT and A25T Cys C variants also showed similar intracellular steady state levels. Furthermore, A25T Cys C did not activate the unfolded protein response and followed the same canonical endoplasmic reticulum (ER)-Golgi trafficking pathway as WT Cys C. WT Cys C has been shown to undergo signal sequence cleavage between residues Gly26 and Ser27. While the A25T polymorphism did not affect Cys C secretion, we hypothesized that it may alter where the Cys C signal sequence is preferentially cleaved. Under normal conditions, WT and A25T Cys C have the same signal sequence cleavage site after Gly26 (referred to as ‘site 2’ cleavage). However, in particular circumstances when the residues around site 2 are modified (such as by the presence of an N-terminal FLAG tag immediately after Gly26, or by a Gly26Lys (G26K) mutation), A25T has a significantly higher likelihood than WT Cys C of alternative signal sequence cleavage after Ala20 (‘site 1’) or even earlier in the Cys C sequence. Overall, our results indicate that the A25T polymorphism does not cause a significant reduction in Cys C secretion, but instead predisposes the protein to be cleaved at an alternative signal sequence cleavage site if site 2 is hindered. Additional N-terminal amino acids resulting from alternative signal sequence cleavage may, in turn, affect the protease inhibition function of Cys C.

## Introduction

Cystatin C (Cys C) is a small, 13.3 kDa reversible competitive inhibitor of papain-like cysteine proteases which is ubiquitously expressed throughout the body, including in the testes, liver, pancreas, brain, and retinal pigmented epithelium (RPE) [1, 2]. Cys C inhibits papain as well as cathepsin-family proteases such as cathepsin B, H, L and S [3]. Cys C binds tightly to these proteases, resulting in dissociation constants in the sub-nanomolar to nanomolar range [3]. Appropriate regulation of proteases in general is of fundamental importance for normal development (reviewed in [4]) and for the prevention of a plethora of diseases ranging from retinal degeneration/vascularization [5] to cancer (reviewed in [6]). Cys C has specifically been shown to be involved in a number of anti-bacterial, anti-viral and anti-amyloid processes (reviewed in [7]) as well as other biological events such as inflammation (reviewed in [7]), cancer [8], and cell proliferation [9]. However, the underlying biological processes that contribute to and regulate Cys C function (and dysfunction) are relatively poorly understood [10].

Two variations in the Cys C coding sequence have been associated with disease. A Leu68Gln (L68Q, also identified as L94Q according to amino acid numbering which includes the typical signal sequence) mutation in Cys C is associated with hereditary cerebral hemorrhage with amyloidosis [10–12], while an Ala25Thr (A25T) polymorphism has been associated with both autosomal recessive late-onset Alzheimer’s disease (AD) [13–15] as well as exudative age-related macular degeneration (AMD) [16, 17]. The L68Q mutation has been shown to be unstable and result in the formation of amyloid/aggregates [18–21], while the mechanism by which the A25T polymorphism influences disease is less well understood. A number of groups have demonstrated that the A25T substitution reduces levels of Cys C in conditioned medium from transfected cells, cerebrospinal fluid, or plasma [22–26], indicating a possible pathogenic effect. However, other evidence argues against the role of A25T polymorphism in disease [27–32].

Interestingly, while the proteases that Cys C binds to are typically localized to the lysosome (reviewed in [33]), Cys C has been found predominately in extracellular fluid including cerebral spinal fluid, serum and urine [34–36]. Secretory proteins are first synthesized in the cytosol and co-translationally translocated across the endoplasmic reticulum (ER) membrane to enter the secretory system [37, 38]. Typical secreted proteins are directed into the ER by a short, hydrophobic N-terminal signal sequence (generally 10–20 resides) (reviewed in [39]). Factors (i.e., signal recognition particles, SRPs) in the cytosol recognize this hydrophobic signal sequence as it emerges from the ribosome and target the nascent polypeptide to the ER through interactions with the SRP receptor. The nascent polypeptide is extruded into the ER through the Sec61 translocon, and in the process, a signal peptidase complex cleaves the signal sequence (reviewed in [40]). Consistent with its extracellular presence, WT Cys C has been predicted (and demonstrated) to have a 26 residue signal sequence [35, 41, 42]. While some groups have postulated that the A25T polymorphism prevents Cys C signal sequence cleavage [22], a serendipitous finding identified A25T Cys C as having an alternative, 20 residue signal sequence as well as an O-linked glycan which likely resides on Ser27 or Ser28, residues close to the normal Cys C signal sequence cleavage site of Gly26 [43]. Given these observations, in combination with the uncertainty of the impact of the A25T polymorphism on Cys C protein homeostasis, we decided to introduce either WT or A25T Cys C into HEK-293T and ARPE-19 cells to develop a further understanding of whether, or how, the AD/AMD-associated polymorphism alters Cys C signal sequence cleavage as well as secretion.

## Materials and Methods

### Plasmid generation

Cystatin C (Cys C, kind gift of Dr. Efrat Levy, New York University Langone Medical Center) was amplified by polymerase chain reaction (PCR) and inserted into the pENTR1A Dual Selection vector (Life Technologies, Carlsbad, CA) using the BamHI and EcoRI restriction sites. Mutations (A25T and G26K) as well as FLAG-tag insertions (DYKDDDDK) were generated by the Q5 Mutagenesis Kit (New England Biolabs (NEB), Ipswich, MA). Cys C constructs were then recombined into either the pTREx-DEST30, pcDNA-DEST40, pcDNA-DEST47, or pAd/CMV/DEST (Life Technologies) destination vector by an LR Clonase II reaction (Life Technologies). All constructs described herein were expressed in a constitutive manner. Only recombination into the pcDNA47-DEST vector altered the encoded Cys C protein. In this instance, we used a pENTR1A Cys C FLAG entry construct without a stop codon. After recombination into the pcDNA-DEST47 vector, the resulting construct encoded for Cys C FLAG green fluorescent protein (GFP), containing a C-terminal FLAG tag followed by a 22 amino acid linker (WNSRPHSRYLDPAFLYKVVRSR) and then a cycle 3 variant of GFP. All constructs were verified by sequencing.

### Cell culture

Human embryonic kidney cells (HEK-293T, Life Technologies) were maintained in Dulbecco’s Modified Eagle Medium (DMEM, Corning, Corning, NY) media supplemented with 10% fetal bovine serum (FBS, Omega Scientific, Tarzana, CA) and penicillin/streptomycin/glutamine (P/S/Q, Corning). Human retinal pigment epithelial cells (ARPE-19, American Type Culture Collection, Manassas, VA) were maintained in DMEM/F12 (Corning) media also with FBS and P/S/Q. All cell lines were cultured at 37°C and 5% CO_2_. Both cell lines were authenticated using short tandem repeat profiling (University of Arizona Genetics Core, Tucson, AZ).

For transfections, HEK-293T cells were plated at a density of 100,000–120,000 cells/well of a 12 well plate and transfected with cytomegalovirus (CMV)-driven constructs using either X-tremeGENE 9 ((Roche, Piscataway, NJ) 1 μg DNA: 3 μL transfection reagent) or X-tremeGENE HP ((Roche) 1 μg DNA: 2 μL transfection reagent), as described previously [44]. For a subset of experiments (n = 3), a humanized *Gaussia* luciferase (hGLuc) construct serving as a control was co-transfected along with Cys C plasmids (50 ng hGLuc: 1 μg of Cys C plasmid) using X-tremeGENE HP. Co-transfection did not change Cys C secretion. For ARPE-19 transfections, two days before the transfection, cells were replated into 10 cm dishes to ensure that they did not reach confluence. The day before the transfection, ARPE-19 cells were replated at a density of 100,000 cells/well of a 12 well plate. Cells were then transfected using Lipofectamine 3000 ((Life Technologies), 1 μg of DNA, 3 μL of Lipofectamine 3000, 1 μL of P3000 reagent). Transfection efficiency was assessed by transfecting an enhanced GFP (eGFP) construct (pEGFP-N1, Clontech, Mountain View, CA) and estimating the number of eGFP-positive cells 24 h after transfection (see S1A–S1F Fig, S2D and S2E Fig).

### Quantitative PCR (qPCR)

Transcript levels of transfected cells were evaluated by extracting cellular RNA (Aurum Total RNA Mini Kit, BioRad, Hercules, CA) followed by generation of the corresponding complementary DNA (cDNA, RealMasterScript, 5 Prime, Gaithersburg, MD) and SYBR Green detection (Power SYBR Green Master Mix, Life Technologies) on a QuantStudio 6 (Life Technologies). Ribosomal protein, large, P2 (RPLP2, housekeeping gene), asparagine synthetase (ASNS, indicative of activating transcription factor 4 [ATF4] activation), glucose-regulated protein 78 (GRP78, indicative of activating transcription factor 6 [ATF6] activation/cleavage) and ER-localized DnaJ homolog 4 (ERdj4, indicative of X-box binding protein 1 [XBP1] splicing) primers were described previously [45]. Cys C qPCR primers were designed using Primer3 software (http://bioinfo.ut.ee/primer3-0.4.0/); Cys C forward primer: 5’-GCGAGTACAACAAAGCCAGC-3’, Cys C reverse primer: 5’-ATGTGGCTGGTCATGGAAGG-3’.

### Signal sequence prediction

SignalP versions 2.0 to version 4.1 (http://www.cbs.dtu.dk/services/SignalP/) were used to predict the likelihood of signal sequence cleavage of the different protein variants described in this paper. The following parameters were used: Organism—Eukaryotes; D-cutoff value—default; Graphics output—PNG and EPS; Output format—standard; and Method—input sequence may include TM regions. This software is based on the following paper: [46].

### Western blotting

For western blots performed on neat, conditioned media, cells were transfected for 24 h, after which media was changed and replaced with the corresponding growth media containing 2% FBS (to minimize eventual gel warping caused by excess bovine serum albumin) for an additional 24 h (therefore, western blot analysis was performed a total of 48 h after the initial transfection). For analysis of secreted proteins, 40 μL aliquots of conditioned media were taken and denatured at 70°C for 10 min using 1x lithium dodecyl sulfate (LDS) buffer with reductant. For western blotting of intracellular proteins, cells were harvested by trypsinization and cells were lysed in RIPA buffer (50 mM Tris, pH 7.4, 150 mM NaCl_2_, 1% Triton-X [v/v], 0.1% sodium dodecyl sulfate (SDS) [w/v], 0.5% sodium deoxycholate [w/v]) with Halt protease inhibitors (Pierce, Rockford, IL) followed by centrifugation. Soluble protein was normalized using a bicinchoninic acid (BCA) assay (Thermo Fisher Scientific, Waltham, MA) and 40 μg of protein was denatured with 1x LDS buffer with reductant. Conditioned media or intracellular lysates were then loaded on a 4–12% BOLT gel and run for 25–30 min at 200 V using 2-(*N*-morpholino)ethanesulfonic acid (MES) buffer. Proteins were transferred to nitrocellulose membranes using a G2 Blotter (Pierce). Blots were blocked in Odyssey Blocking Buffer (LI-COR, Lincoln, NE). After blocking, blots were probed with an anti-Cys C antibody (1:500, Pierce), anti-FLAG M2 (1:2000, Sigma, St. Louis, MO), or anti-β-actin (1:10,000, Sigma or 1:2000, LI-COR) followed by an appropriate infrared-conjugated secondary antibody (1:10,000, LI-COR). All blots were imaged and quantified on a LI-COR Odyssey Fc (LI-COR).

### Immunoprecipitation (IP)

HEK-293T cells were transfected as described above for 24 h. Media was changed 24 h after transfection with 10% FBS-containing media and allowed to condition for an additional 24 h. Media was removed and an equal amount (400 μL) was immunoprecipitated (IP’d) for ≥2 h at 4°C using 3–5 μL of either anti-FLAG M1 agarose beads (Sigma) or anti-FLAG M2 magnetic beads (Sigma). Beads were washed thrice with Hanks Balanced Salt Solution with Ca^+2^ (HBSS, Sigma) and eluted in 1x LDS buffer without reductant. After the elution, when the sample was removed from the beads, reductant was added, and the sample was further denatured. Samples were run for western blotting as described earlier.

### Mass spectrometry (MS)

As an orthogonal approach to determine Cys C signal sequence cleavage, we validated the IP and western blotting approach using intact mass spectrometry (MS) on purified Cys C FLAG variants. Briefly, ARPE-19 cells (2–3 million cells per 10 cm dish) were infected with adenovirus (produced as described previously [47]) encoding for each of the C-terminal FLAG-tagged Cys C constructs for 48–72 h followed by a media change (24 h) and IP with anti-FLAG M2 magnetic beads (10 μL, ≥2 h at 4°C). Beads were washed thrice in HBSS and the FLAG protein was eluted using 1x FLAG peptide (Sigma). A portion of the eluant was analyzed by SDS-PAGE followed by Coomassie Blue staining, while the remainder of the protein was submitted for intact MS analysis (University of Texas Southwestern Proteomics Core, Dallas, TX). Briefly, protein samples were directly injected onto a 1.0 mm x 2 cm guard column, packed with POROS R1 20 μm reversed-phase media (Applied Biosystems, Foster City, CA). After desalting, the proteins were eluted onto an Agilent 6540 UHD Accurate-Mass Q-ToF mass spectrometer. The acquired mass spectra were deconvoluted using the maximum entropy deconvolution algorithm in the Agilent MassHunter software in order to obtain the molecular weight.

### Viability

The resazurin assay [48] which reports on mitochondrial reduction potential, was employed to assess potential differences in cell viability due to transfection of different Cys C variants and/or cell density differences after plating. This assay was performed essentially as described previously [49], but samples were incubated for 1 h, and the fluorescence was read on a Synergy II plate reader (BioTek, Winooski, VT).

## Results

### Cys C secretion and intracellular steady state levels in transfected HEK-293T cells

**Fig 1:**
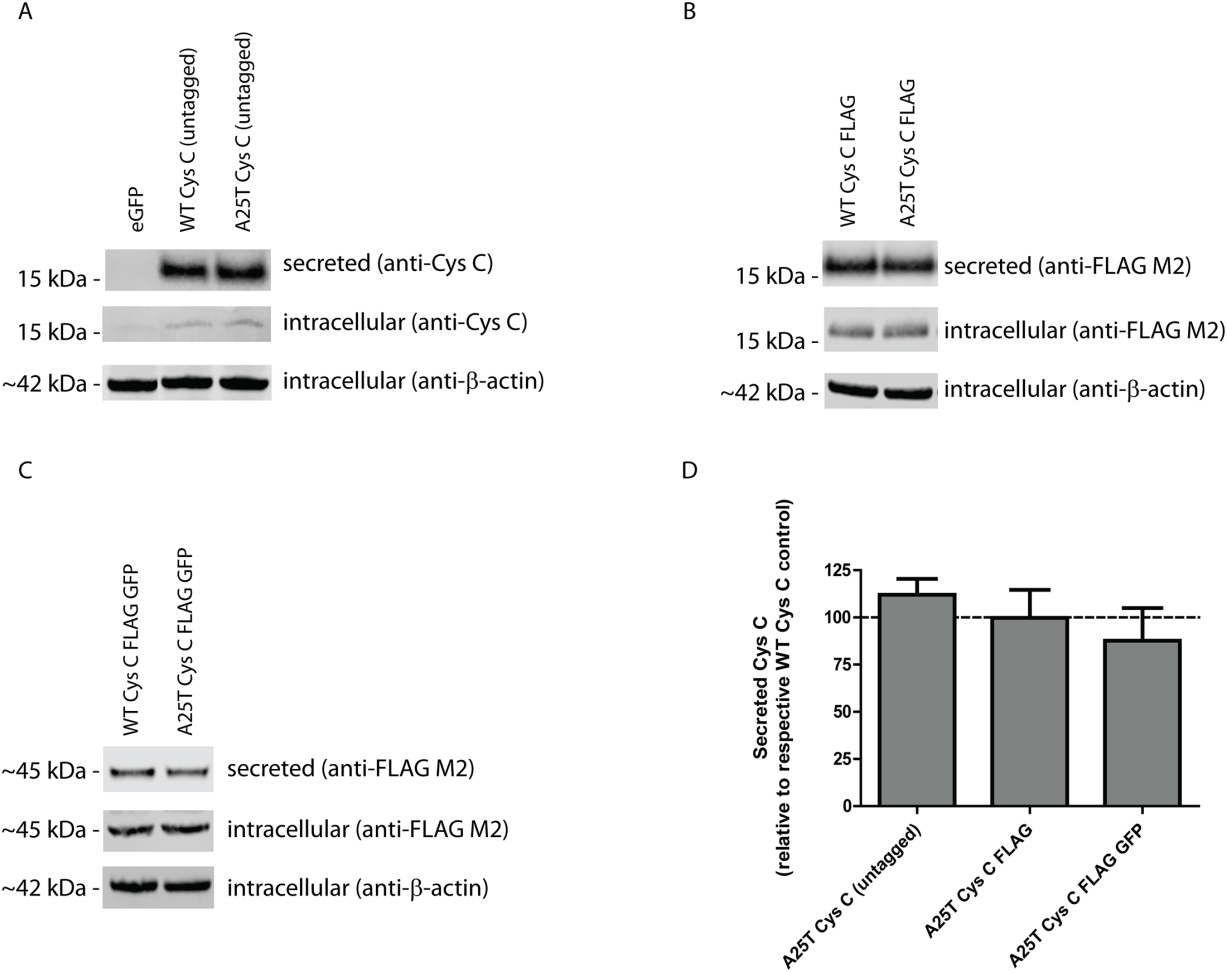
A25T Cys C is secreted as efficiently as WT Cys C from HEK-293T cells. (A-C) HEK-293T cells were transfected with (A) untagged WT or A25T Cys C constructs, (B) WT or A25T Cys C FLAG constructs, or (C) WT or A25T Cys C FLAG GFP constructs. Forty-eight hours post transfection, conditioned media (40 μL) or cell lysates (40 μg) were run under denaturing, reducing conditions (with lithium dodecyl sulfate and DTT) on a 4–12% BOLT gel. Proteins were transferred to nitrocellulose membranes, probed with an anti-Cys C (1:500, Pierce), anti-FLAG M2 (1:2000, Sigma) or anti-β-actin (1:10,000, Sigma, or 1:2,000, LI-COR) antibody followed by an infrared-conjugated secondary antibody (1:10,000, LI-COR), representative data of at least 3 independent experiments. (D) Secreted protein bands were quantified using LI-COR software and data was presented as A25T Cys C secretion levels as percent of each respective WT Cys C control. n ≥ 7, mean ± S.D.

Given its potential importance in exudative AMD and late-onset AD progression, we decided to extensively characterize the secretion and intracellular levels of WT and A25T Cys C. Initially, we generated three pairs of Cys C constructs; one encoding for untagged WT or A25T Cys C, one encoding for WT or A25T Cys C with a C-terminal FLAG tag (Cys C FLAG), and one set of constructs encoding for WT or A25T Cys C with a C-terminal FLAG and GFP-tag (Cys C FLAG GFP). After transfection of these constructs into HEK-293T cells, we monitored the secretion and intracellular steady state levels of each Cys C variant (Fig 1A–1C). Surprisingly, we found that each pair of Cys C proteins (i.e. untagged, FLAG-tagged, or FLAG GFP-tagged) were secreted similarly. Untagged A25T Cys C was secreted at 112 ± 9% of untagged WT Cys C; A25T Cys C FLAG was secreted at 100 ± 15% of WT Cys C FLAG levels; and A25T Cys C FLAG GFP was secreted at 88 ± 17% of WT Cys C FLAG GFP levels (Fig 1D). Furthermore, intracellular steady state levels of either WT or A25T Cys C were consistently similar (Fig 1A–1C). To further demonstrate that these similarities in WT and A25T Cys C were not be due alterations in transfection efficiency, viability, or media sampling, we performed a series of additional supporting experiments. Transfection efficiency into HEK-293T cells was consistent and routinely above 70% (S1A–S1F Fig). Co-transfection of an additional secreted protein, humanized *Gaussia* luciferase (hGLuc) along with Cys C demonstrated that there were similar hGLuc levels in WT and A25T Cys C transfected samples, indicating that our transfections and sampling of the conditioned media were consistent (S2A Fig). Transcript levels between WT and A25T Cys C for each set of variants did not differ significantly amongst any of the variants (S2B Fig). Finally, viability of WT and A25T Cys C-expressing cells were nearly identical across the different Cys C versions (S2C Fig).

### Cys C secretion, trafficking and the unfolded protein response in ARPE-19 cells

**Fig 2:**
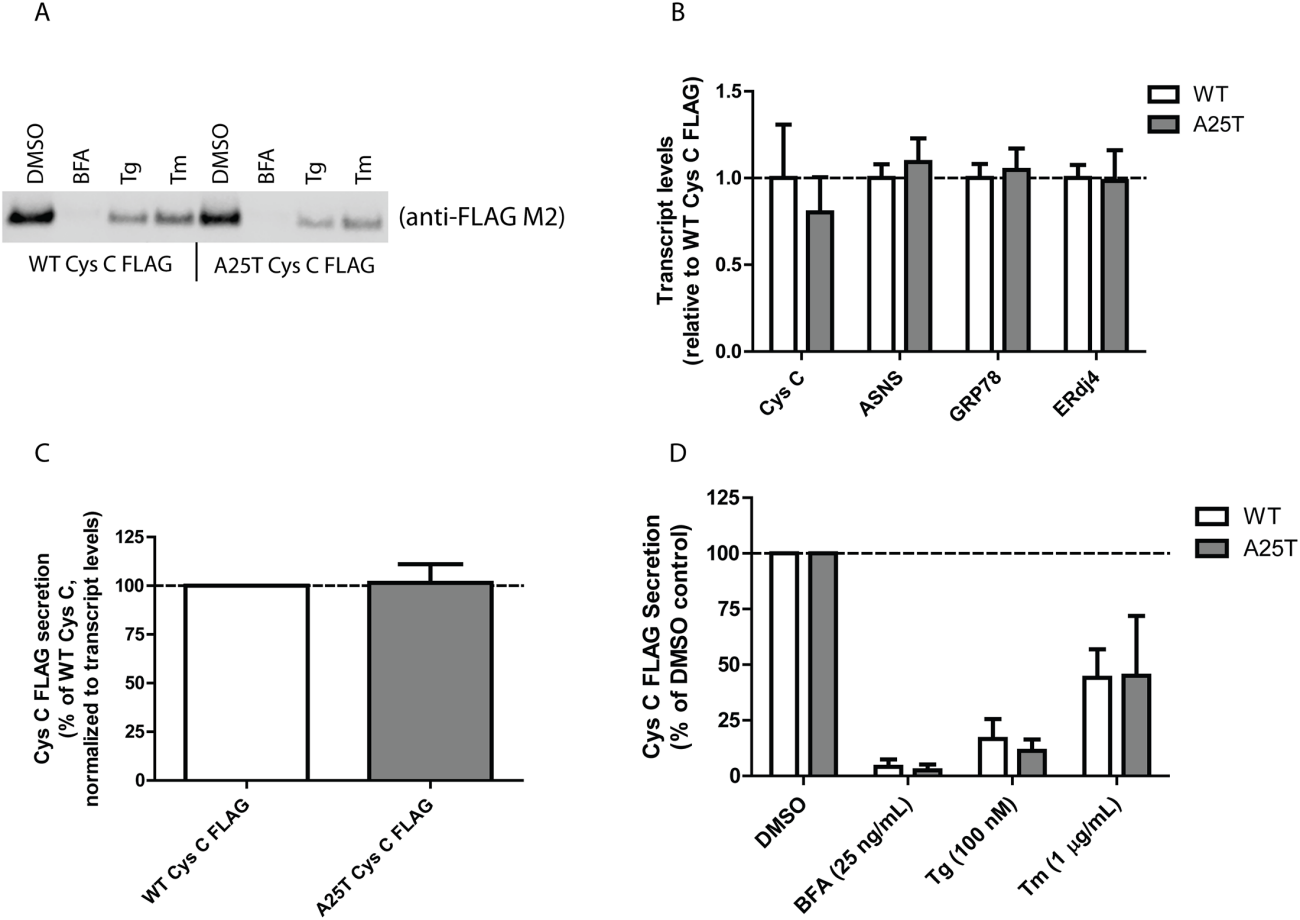
A25T Cys C is readily secreted from ARPE-19 cells. (A) ARPE-19 cells were transfected with WT Cys C FLAG or A25T Cys C FLAG constructs. Forty-eight hours post transfection, cells were then treated with the indicated ER stressor (BFA = brefeldin A; Tg = thapsigargin; Tm = tunicamycin) or vehicle control (DMSO) for 24 h. 40 μL of conditioned media was then analyzed by western blotting using the anti-FLAG M2 antibody. (B) qPCR analysis of transfected ARPE-19 cells for changes in Cys C and unfolded protein response (UPR)-dependent transcripts (mean ± 95% C.I.). (C) Secretion of WT and A25T Cys C (DMSO treatment) was quantified by LI-COR and normalized for any differences in transcription based on the corresponding qPCR results (mean ± S.D.). (D) LI-COR quantification of secreted Cys C after drug treatments (mean ± S.D.). Representative data are shown for 3 independent experiments for A, and n ≥ 3 for panels B-D.

While HEK-293T cells are easy to manipulate genetically and have served as model systems in numerous studies, human retinal pigment epithelium cells (ARPE-19) would arguably be a better system to test potential secretion differences between WT and A25T Cys C from the perspective of AMD. Therefore, we transfected WT and A25T Cys C FLAG constructs into ARPE-19 cells and followed Cys C secretion (Fig 2A). Transfection efficiency was routinely above 65% (S2D and S2E Fig). There was a slight reduction (by ~20%) in A25T Cys C secretion compared to WT Cys C (Fig 2A), however, this difference was due to reduced expression levels of A25T (Fig 2B). Therefore, we quantified Cys C secretion and normalized those levels according to any differences in transcription. After accounting for these transcriptional differences, A25T Cys C was secreted at 102 ± 10% of WT Cys C levels (Fig 2C), a result similar to those we obtained in HEK-293T cells (100 ± 15%, Fig 1B and 1D). We next verified that both WT and A25T Cys C followed the same canonical ER-Golgi secretion pathway, and not nonclassical (or ER/Golgi independent) secretion pathways (reviewed in [50]). Transfected ARPE-19 cells were treated with low levels of brefeldin A (BFA), thapsigargin (Tg) or tunicamycin (Tm) for 24 h. As expected for a secreted protein that follows conventional ER-Golgi secretion pathways, blocking ER to Golgi trafficking with BFA all but eliminated both WT and A25T Cys C secretion (Fig 2A and 2D, 4 ± 3% of vehicle-treated WT Cys C levels, and 3 ± 3% of vehicle-treated A25T Cys C levels). Furthermore, inducing ER stress by altering ER calcium influx with Tg or inhibiting N-linked glycosylation with Tm substantially reduced both WT and A25T Cys C levels similarly (Fig 2A and 2D). While our results up until this point indicate that WT and A25T Cys C behave similarly, we nonetheless explored the possibility that A25T Cys C may be misfolded in some regard and triggers ER stress. In order to do so, we monitored the expression of genes downstream of each of the UPR signaling pathways (Fig 2B, ASNS is downstream of ATF4 upregulation [51], GRP78 is downstream of ATF6 cleavage [52], and ERdj4 is downstream of XBP1 splicing [52]). We could detect no substantial increase in activation of any of the arms of the UPR in response to A25T Cys C expression. As demonstration that expression of these genes are indeed indicative of UPR activation, we performed additional qPCR experiments ARPE-19 cells using the ER stressors mentioned above (S2F Fig). Overall, these data, taken together with those from Fig 1, suggest that WT and A25T Cys C behave similarly regarding their extent of secretion and utilize the same cellular pathways for exiting the cell.

### Cys C has two potential signal sequence cleavage sites

**Fig 3:**
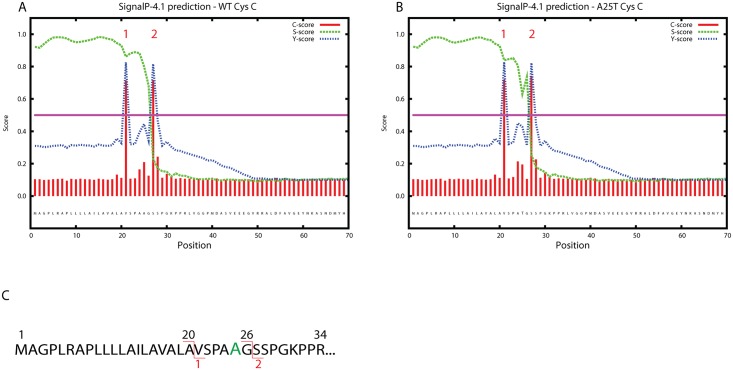
Signal sequence prediction of WT and A25T Cys C using SignalP 4.1 software. (A, B) WT and A25T Cys C amino acid sequence was analyzed by SignalP 4.1 software and the resultant predicted cleavage sites, designated as site 1 and site 2, are shown. (C) In WT Cys C, site 1 cleavage occurs between Ala20 and Val21, site 2 cleavage occurs between Gly26 and Ser 27. The Ala residue in the A25T mutation is enlarged and colored green.

Since we did not observe any obvious differences in WT or A25T Cys C secretion in either HEK-293T or ARPE-19 cells, we further scrutinized how, or whether, the A25T polymorphism could alter Cys C processing or function. Previously, the 26 residue WT Cys C signal sequence (predicted in [35]) was verified by mass spectrometry [41] and Edman degradation [42]. However, in 2009, a study wherein researchers enriched for O-glycosylated proteins isolated from cerebrospinal fluid, found that the A25T Cys C polymorphic variant showed evidence of an alternatively cleaved signal sequence corresponding to a 20 residue signal sequence [43]. Given these intriguing results, we ran the primary amino acid sequence of WT Cys C through sequence prediction software, SignalP [46]. The most recent version of this software (version 4.1) predicts that WT Cys C has two potential cleavage sites, each of which is predicted to have nearly identical propensities for cleavage; one site is after Ala20 (designated as site 1), and another site is after Gly26 (designated as site 2, Fig 3A and 3C). This ‘two site’ characteristic appears to be unique amongst other cystatin proteins including cystatin A, D, F, M and S (S3 Fig). The SignalP 4.1 software did not predict that the A25T polymorphism alone would change the signal sequence cleavage site substantially (Fig 3B). However, while the most recent version of the signal sequence software predicted cleavage after Ala20 for WT and A25T Cys C (S1 Table), previous prediction software versions proposed a cleavage site between Gly26 and Ser27 (SignalP 2.0 and 3.0, S1 Table). Thus, according to this software, the proposed Cys C signal sequence cleavage site of WT or A25T Cys C could occur at either site 1 or at site 2.

### Modification of residues surrounding site 2 influences signal sequence cleavage

**Fig 4:**
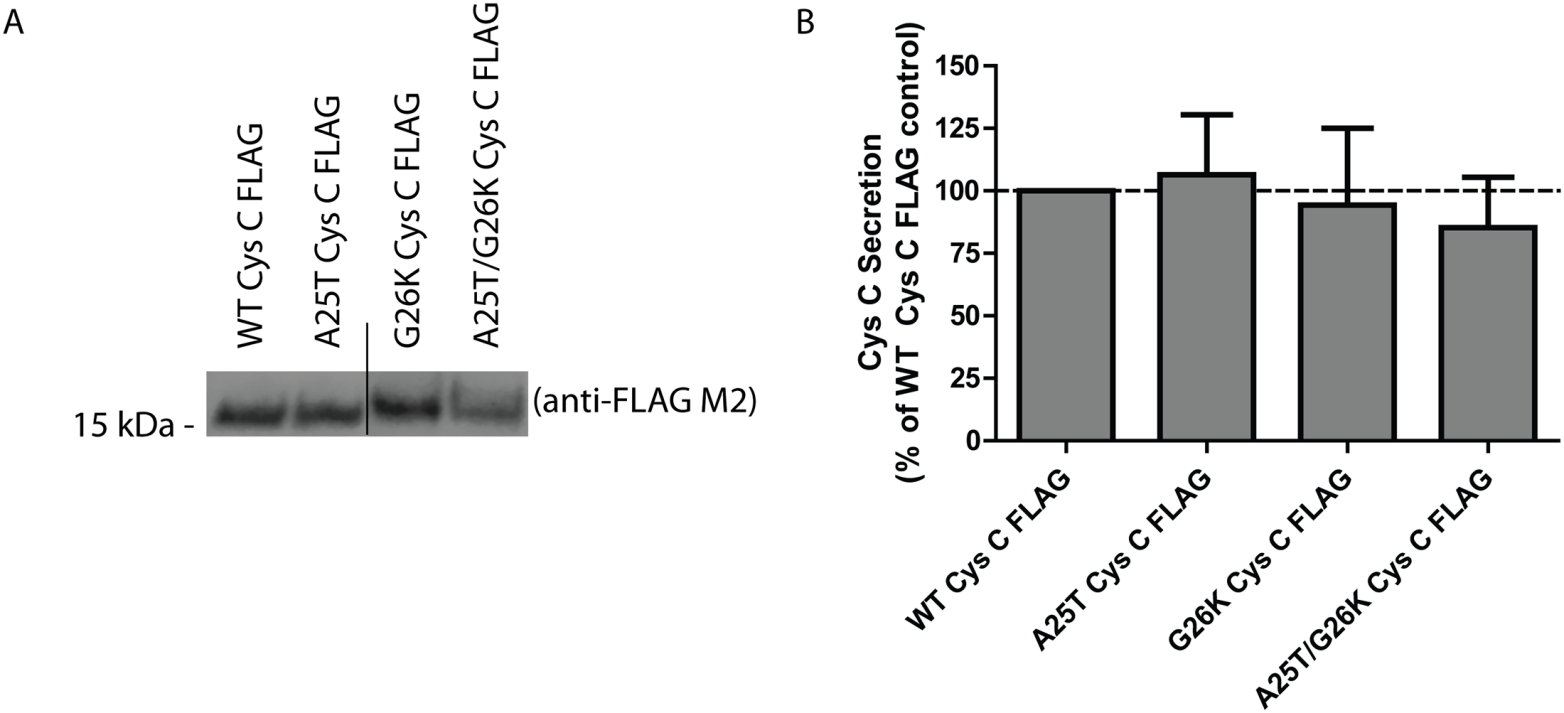
A G26K mutation eliminates Cys C signal sequence cleavage at site 2, and favors site 1 cleavage. (A) WT, A25T, G26K or G26K/A25T Cys C FLAG constructs were transfected into HEK-293T cells and conditioned media samples were analyzed by western blotting. Representative data are shown for >3 independent experiments. (B) LI-COR quantification of secreted levels of Cys C variants listed in (A), n ≥ 3, mean ± S.D.).

To test whether Cys C could in fact be preferentially cleaved at site 1 (an alternative cleavage site) vs. site 2 (the canonical site experimentally determined by previous studies), we altered the Cys C signal sequence by a simple point mutation; a positively charged lysine residue at the ‘-1’ position (Gly26Lys, G26K) immediately preceding the site 2 cleavage site. Charged amino acids are strongly disfavored at this position [53], and would be predicted to disrupt signal sequence cleavage (S4A and S4B Fig), preventing site 2 cleavage and instead favoring site 1. Indeed, we observed that modification of the signal sequence in this manner, while not substantially impacting the amount of Cys C secretion, increased its apparent molecular weight, which is likely indicative of alternative signal site cleavage (Fig 4A and 4B). We did note, however, that A25T/G26K Cys C FLAG consistently showed a more diffuse banding profile when compare to other variants (Fig 4A). Cells expressing these variants of Cys C showed no signs of alterations in viability nor Cys C expression levels (S5A and S5B Fig).

While the presence of charged residues such as lysine can increase the apparent molecular weight of Cys C [54], the Lys26 residue would be located between the two signal sequence cleavage sites. Therefore, the observation that the G26K mutation alters the apparent molecular weight of Cys C indicates that Lys26 is likely present in the secreted protein. If the G26K mutation would not alter the canonical signal sequence cleavage site, then the resulting secreted protein (which would not include Lys26) would be expected to have a molecular weight similar to WT or A25T Cys C. Thus, we hypothesize that Cys C is capable of undergoing site 1 signal sequence cleavage only if site 2 cleavage is eliminated or reduced. Under ‘normal’ conditions, WT and A25T Cys C are cleaved at site 2. However, upon disruption of site 2, either by mutation or potentially post-translational modifications near site 2, site 1 can be used.

### The A25T polymorphism increases the likelihood of alternative Cys C signal sequence cleavage

**Fig 5:**
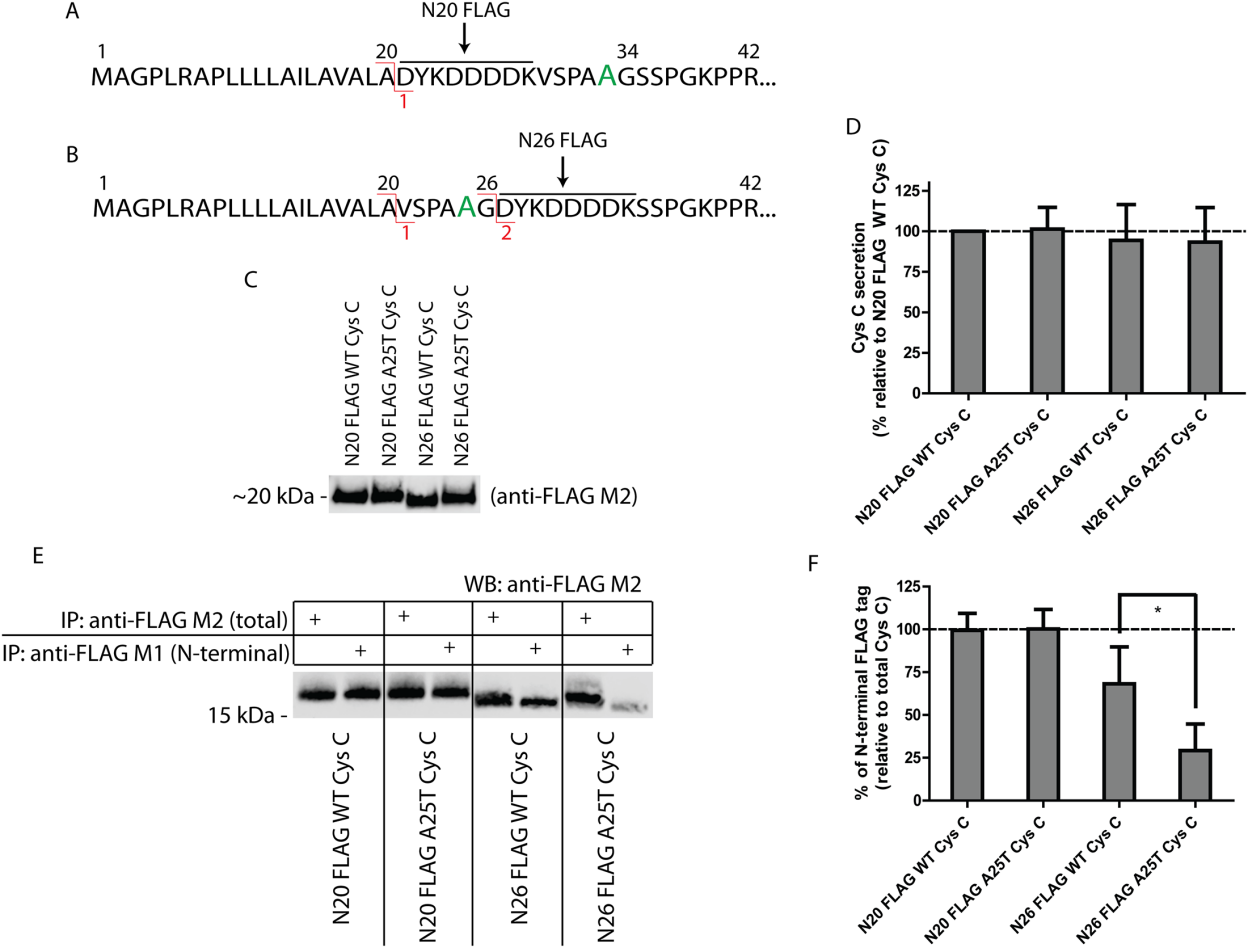
The A25T polymorphism predisposes Cys C to alternative signal sequence cleavage. (A) A FLAG tag after residue 20 (N20 FLAG) is predicted to eliminate site 2 cleavage. (B) However, a FLAG tag after residue 26 (N26 FLAG) is predicted to keep the two potential cleavage sites. (C) Secretion of N-terminal FLAG Cys C variants is similar. The secretion of N-terminal FLAG Cys C proteins (either N20 FLAG; FLAG tag after Ala20, or N26 FLAG; FLAG tag after Gly26) was assessed after plasmid transfection into HEK-293T cells. Representative data of at least 6 experiments. (D) Quantification of FLAG Cys C secretion in (C) by LI-COR software. n ≥ 6, mean ± S.D. (E) Differential IP of secreted FLAG Cys C from HEK-293T cells using either anti-FLAG M1 or anti-FLAG M2 beads. (F) Quantification of the relative levels of N-terminal (anti-FLAG M1 IP) to total (anti-FLAG M2 IP) FLAG-tagged protein. n ≥ 3, mean ± S.D., * = p < 0.05 by a paired t-test.

The G26K experiments demonstrated that Cys C can be altered such that site 1 cleavage is favored at the expense of site 2. To further probe whether the A25T polymorphism could influence signal sequence cleavage in particular scenarios when the sequence around site 2 is altered or modified, thus shifting the preference of Cys C signal sequence cleavage from site 2 to site 1, we designed a series of N-terminal FLAG-tagged Cys C constructs. Placement of the FLAG octapeptide immediately after site 1 (Ala20), designated as N20 FLAG Cys C, is predicted to retain site 1 cleavage, but eliminate the possibility of site 2 cleavage (Fig 5A, S4C Fig). Positioning of the FLAG tag after site 2 (Gly26), designated as N26 FLAG Cys C, is predicted to retain both potential cleavage sites, but could serve as a disrupting force in signal sequence cleavage due to its abundance of charged and aromatic residues (Fig 5B, S4D Fig). We next developed an approach that utilizes unique properties of two separate anti-FLAG antibodies. The anti-FLAG M1 antibody recognizes the FLAG peptide only when it is positioned at the extreme N-terminus of a protein. The anti-FLAG M2 antibody, in contrast, is able to recognize the FLAG peptide regardless of its positioning within a protein. Initially, we introduced both the N20 and N26 FLAG Cys C variants into HEK-293T cells and monitored the secretion of Cys C in the conditioned media by Western blotting using the anti-FLAG M2 antibody (Fig 5C). All variants were readily secreted from the transfected cells and were present in the conditioned media at similar levels (Fig 5C and 5D). N20 FLAG A25T Cys C was secreted at 101 ± 13% of N20 FLAG WT Cys C, while N26 FLAG WT Cys C and N26 FLAG A25T Cys C were secreted at 94 ± 22% and 93 ± 21% of N20 FLAG WT Cys C levels, respectively (Fig 5D). These studies demonstrated the total amount of secreted protein was similar across all variants (which was also corroborated by viability and transcriptional studies, S5C and S5D Fig), but they did not yield definitive insight into how the Cys C signal sequence was processed (although a slight increase in the apparent molecular weight of N26 FLAG A25T Cys C hints at site 1 cleavage, Fig 5C).

To more definitively identify and quantify the cleavage site propensity for each of the Cys C variants, we immunoprecipitated (IP’d) equal amounts of conditioned media from Cys C transfected cells with either anti-FLAG M1 or anti-FLAG M2-conjugated beads. The amount of Cys C that is pulled down by the anti-FLAG M1 antibody represents the amount of protein with an extreme N-terminal FLAG tag, whereas the amount of Cys C pulled down by the anti-FLAG M2 antibody represents total FLAG-tagged Cys C. We found that the entirety of the N20 FLAG WT Cys C (99 ± 10%) or N20 FLAG A25T Cys C (100 ± 11%) that was secreted harbored an extreme N-terminal FLAG tag (Fig 5E and 5F). These results suggest that if site 2 cleavage is prevented, both WT and A25T Cys C can still be cleaved at site 1 and efficiently secreted. Application of a similar IP approach with secreted N26 FLAG Cys C variants (which have the ability to be cleaved at site 1 or 2) demonstrated that the A25T polymorphism significantly decreased the likelihood of site 2 cleavage compared to N26 FLAG WT Cys C. On average, 71% of the N26 FLAG A25T Cys C was not cleaved at site 2, whereas 32% of the N26 FLAG WT Cys C protein was not cleaved at site 2 (Fig 5E and 5F).

### Mass spectrometry (MS) confirmation of Cys C signal sequence cleavage

**Fig 6:**
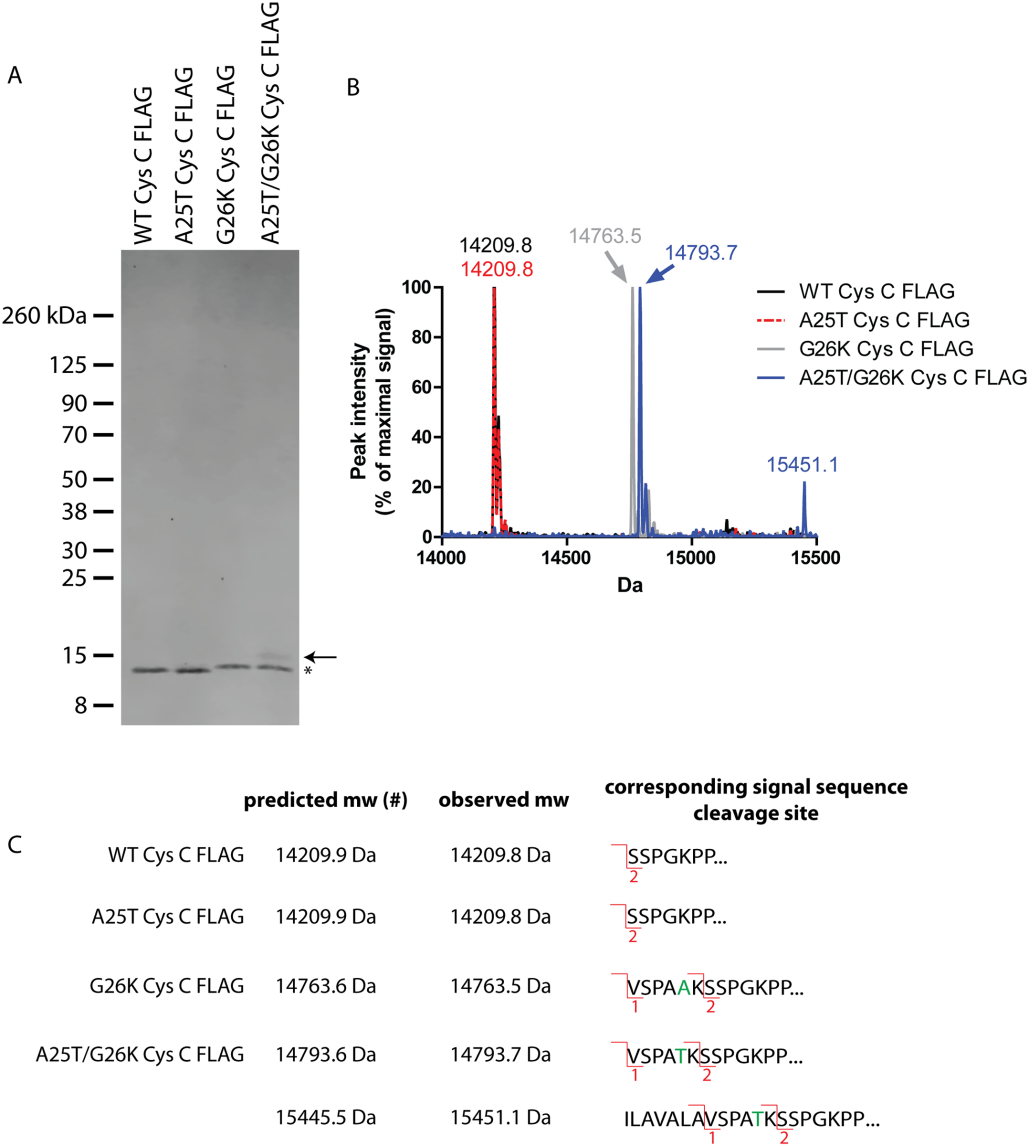
Mass spectrometry (MS) confirmation of Cys C signal sequence cleavage sites. (A) ARPE-19 cells were infected with adenovirus encoding for the designated Cys C FLAG variants and the amount of secreted Cys C was isolated by IP using anti-FLAG M2 beads. Cys C was eluted using the FLAG peptide and run on an SDS-PAGE gel followed by Coomassie Blue staining. Asterisk denotes site 1 cleavage products, whereas the arrow denotes a newly identified cleavage product only found in A25T/G26K Cys C. (B) Intact MS of eluted Cys C purified from ARPE-19 conditioned media. Eluted, IP’d Cys C FLAG variants (not separated by SDS-PAGE) were analyzed by intact MS. Numbers above each peak denote molecular weight in Da. (C) Comparison of predicted and observed molecular weights and the corresponding predicted signal sequence cleavage site. (#) Note: the predicted molecular weight was based on assuming a loss of 4 Da due to two Cys C disulfide bonds and the loss of the C-terminal lysine (128 Da) during the purification process [55].

As an alternative approach to the biochemical antibody recognition assay described in Fig 5, we also used intact MS to confirm the cleavage sites of WT, A25T, G26K and A25T/G26K Cys C FLAG (bearing a C-terminal FLAG tag) proteins isolated from ARPE-19 cells. To achieve high levels of secreted Cys C, we infected ARPE-19 cells with adenovirus encoding for the different Cys C variants and then purified the secreted protein by IP. After elution with the FLAG peptide, we were able to recover pure Cys C FLAG protein in the absence of other detectible proteins (Fig 6A). The major Cys C FLAG species in each lane migrated as predicted based on our previous results in HEK-293T cells (Fig 4) with the G26K variants migrating at a slightly higher apparent molecular weight (Fig 6A, asterisk). However, one important difference that we noted in these experiments was the presence of a small amount of additional cleavage product (~15.5 kDa) in the A25T/G26K Cys C FLAG lane (Fig 6A, arrow). We then submitted a portion of the eluted Cys C protein (which was not separated by SDS-PAGE) for intact MS analysis. As predicted, we found that WT and A25T Cys C FLAG had overlapping chromatograms with a major species at 14209.8 Da (Fig 6B). After accounting for the two disulfides present in Cys C (- 4 Da), and the loss of the C-terminal lysine of the FLAG tag (-128 Da) during production/purification [55], this species corresponds to site 2 cleavage of Cys C (Fig 6C). No site 1 cleavage was detected in either the WT or A25T Cys C samples. The prominent species in both G26K and A25T/G26K Cys C samples were 14763.5 and 14793.7 Da, respectively (Fig 6B). The molecular mass of these samples was different by 30 Da due to the A25T alteration. The observed molecular weights of these variants correspond to site 1 cleavage (Fig 6C) after factoring disulfide bonding and lysine loss. In contrast to WT and A25T Cys C, no site 2 cleavage was detected in either the G26K or A25T/G26K Cys C samples. Arguably, the most interesting result from these studies was the appearance of an additional 15451.1 Da species in the A25T/G26K Cys C FLAG sample (Fig 6B). This species has not previously been identified, but according to its molecular weight, it may be a result of cleavage after Ala13, although the predicted and expected mass are disparate by 5.6 Da (Fig 6C). Interestingly, we did not observe this specific species in the conditioned media from HEK-293T cells transfected with A25T/G26K Cys C FLAG (Fig 4A), which indicates a potential difference in how cells of different origins process this variant.

## Discussion

Herein we presented a comprehensive evaluation of WT and A25T Cys C signal sequence cleavage and secretion from transfected, immortalized cells. Much to our surprise, we found that WT and A25T Cys C were both secreted efficiently from HEK-293T and ARPE-19 cells. Furthermore, A25T Cys C did not trigger the UPR, and followed the same trafficking pathway as WT Cys C. Signal sequence prediction software identified two potential Cys C signal sequence cleavage sites, one after Ala20 (site 1), and another after Gly26 (site 2). While site 2 is the default cleavage site, WT and A25T Cys C are able to be cleaved at either site 1 or 2 and still be secreted efficiently. We noted that when modifications are made near site 2, such as the mutation of Gly26 to lysine (G26K), or the addition of an N-terminal FLAG tag after residue 26 (N26 FLAG), the A25T polymorphism increased the likelihood of alternative signal sequence cleavage either at site 1, or potentially after Ala13. Ultimately, this alternative cleavage results in additional N-terminal amino acids in the mature, secreted protein. These additional N-terminal amino acids may, in turn, affect the protease function of Cys C.

Our findings which demonstrate that WT and A25T Cys C have similar steady state levels of secreted and intracellular protein differ from a number of studies which focused on A25T Cys C secretion [22, 56] or its presence in the plasma/cerebrospinal fluid [24–26]. The reason behind these discrepancies is not clear. In our study, we controlled for potential differences in Cys C expression, Cys C-driven viability changes, and changes in the secretion of proteins other than Cys C (hGLuc). None of these aspects differed significantly between WT Cys C or A25T Cys C-expressing cells. One difference between our study and those that were mentioned previously is that previous studies used Cys C which originated from patients, primary cells or patient-derived cells. Our study used standard, immortalized, cultured cells. Could there be differences in how Cys C is handled in immortalized vs. primary cells/tissue? While the answer to that question is also unknown, ultimately, these discrepancies may be due simply to differences in the complexities of the systems used to study Cys C secretion (i.e., controlled and simple cell culture conditions vs. more complex biological solutions or cells).

Interestingly, we are not the first group to observe that WT and A25T Cys C are present in fluids at similar levels. In agreement with our findings, Chuo *et al*. noted that homozygosity for the A25T polymorphism still conferred significant risk for the development of late-onset AD even though levels of plasma A25T were not significantly lower when compared to WT Cys C in other AD patients [57]. Additionally, it is important to note that six independent studies have disputed the association of the A25T polymorphism with AD in Chinese [27], Italian [28, 29], Japanese [30], Finnish [31] and Dutch [32] populations. Furthermore, one recent meta-analysis study suggested that there is a significant association with the A25T polymorphism and AD in Caucasians, but not Asians [58]. Thus, it appears that the impact of the A25T polymorphism on late-onset AD is not clear-cut, and that the polymorphism may only have an impact in certain ethnic populations or under certain biological situations. While there are a number of studies which probe the role of the A25T Cys C polymorphism in AD [13–15, 27–32], there is a dearth of knowledge regarding its involvement in exudative AMD; only two clinical studies so far have linked the A25T Cys C polymorphism with increased likelihood of exudative AMD [16, 17], one of which utilized metadata from the initial study [17]. Given these observations, some of which show an association between the A25T polymorphism and disease, and some of which do not, combined with the contrasting findings of the impact of the polymorphism on Cys C secretion, we strongly advocate for developing a deeper understanding of the cellular facets which regulate Cys C secretion and function.

Our data suggest that the impact of the A25T polymorphism on Cys C signal sequence and secretion under ‘normal’ conditions is undetectible; A25T Cys C is secreted at similar levels to WT Cys C, does not trigger canonical ER stress pathways, and is cleaved at the canonical, default signal sequence cleavage site, site 2. However, by appending an eight residue FLAG tag, we were able to detect significant differences in how the A25T Cys C signal sequence was processed compared to WT Cys C; A25T had a significantly higher likelihood of site 1 cleavage (71%) compared to WT Cys C (32%). While site 1 cleavage has not been described before for WT Cys C, Nilsson *et al*. detected that A25T Cys C enriched from cerebral spinal fluid was O-glycosylated at Ser27 or Ser28 and still maintained part of the Cys C signal sequence (cleaved at site 1, [43]). We believe that this was not a spurious observation since O-glycosylation software also predicts that A25T Cys C has an increased likelihood of O-glycosylation on Ser28 compared to WT Cys C (http://www.cbs.dtu.dk/services/NetOGlyc/, [59]). However, O-glycosylation occurs primarily in the Golgi apparatus, which is downstream of co-translational protein import into the ER and signal sequence cleavage (reviewed in [60]). Thus, presumably, the decision of where to cleave the Cys C signal sequence occurs before the cell has had a chance to O-glycosylate A25T Cys C. These principles argue against O-glycosylation directly influencing signal sequence cleavage, but suggest, rather, that under certain, as-of-yet unknown conditions, A25T Cys C first undergoes site 1 cleavage, and then the five extra N-terminal amino acids makes Cys C a better O-glycosylation substrate as it is trafficked through the Golgi. Nonetheless, we speculate that the N26 FLAG tag near site 2 or the G26K mutation reveals an otherwise obscured inherent propensity for A25T Cys C to be alternatively cleaved at site 1.

It is unclear whether alternative signal sequence cleavage resulting in additional amino acid residues would affect Cys C protease inhibition function. Previous work by Abrahamson *et al*. and Wallin *et al*. demonstrated that the first ten N-terminal amino acid residues of Cys C were integral for effective inhibition of cathepsin B and L and for internalization into cells [54, 61]. Based on these results, the authors of the former paper suggested that N-terminal amino acids bound to the substrate-binding pocket of cathepsin B and L. We therefore speculate that additional N-terminal amino acids could potentially disrupt cathepsin B/L inhibition and/or prevent cellular reuptake of Cys C, thus reducing levels of functional Cys C. Such an impact on Cys C function could have dramatic effects on Cys C-related cellular processes. While future studies will be dedicated to understanding under what biologic conditions Cys C undergoes alternative signal sequence cleavage, as well as determining the extent of alternative signal sequence cleavage, ultimately, we must first determine whether/how alternative signal sequence cleavage alters Cys C’s protease inhibition function.

## Supporting Information

S1 Fig Representative transfection efficiency using HEK-293T cells. (A-F) HEK-293T cells were transfected with pEGFP-N1 (A, B), WT Cys C FLAG GFP (C, D), or A25T Cys C FLAG GFP (E, F) and imaged 24 h afterward. Bright field (A,C,E) and GFP-channel (B,D,F) images were captured. Transfection efficiencies for HEK-293T cells were consistently above 70%.
(PDF)

S2 Fig Control experiments supporting Figs 1 and 2. (A) Co-expression of a humanized, secreted *Gaussia* luciferase (hGLuc) construct along with Cys C demonstrates similar levels of another secreted protein after transfection. n = 3, mean ± S.D. (B) Cys C expression levels in HEK-293T cells 48 h after transfection. Representative data of at least three independent experiments. Mean ± 95% C.I. (C) Viability comparison of Cys C-expressing HEK-293T cells 48 h after transfection. n ≥ 3, mean ± S.D. (D, E) Representative transfection efficiency of ARPE-19 cells. ARPE-19 cells were transfected with pEGFP-N1 and imaged 24 h later using transmitted light (D) or a GFP filter set (E). Transfection efficiencies for ARPE-19 cells were consistently above 65%. (F) qPCR validation of UPR induction after BFA, Tg or Tm treatment of ARPE-19 cells. Representative data of three independent experiments, mean ± 95% C.I.
(PDF)

S3 Fig Cys C has a unique signal sequence compared to other cystatin proteins. (A) SignaIP 4.1 prediction of cystatin A (a non-secreted cystatin), (B) cystatin C, (C) cystatin D, (D) cystatin F, (E) cystatin M and (F) cystatin S.
(PDF)

S4 Fig Placement of a FLAG tag or mutation of Cys C alters the predicted signal sequence cleavage sites according to SignalP 4.1. (A, B) SignaIP 4.1 prediction of G26K (A) or A25T/G26K Cys C (B). (C) SignaIP 4.1 prediction of N20 FLAG WT Cys C, which eliminates site 2 cleavage. (D) SignaIP 4.1 prediction of N26 FLAG WT Cys C, which maintains the two potential sites. Mutation of Ala25 to Thr did not change the predicted cleavage sites.
(PDF)

S5 Fig Viability and transcriptional analysis of Cys C-expressing HEK-293T cells. (A, C) Viability of the transfected HEK-293T cells was evaluated 48 h post transfection by the resazurin assay. (B, D) Cys C expression levels were monitored by qPCR 48 h after transfection. n ≥ 3, mean ± S.D. for all panels.
(PDF)

S1 Table Predicted Cys C signal sequence cleavage sites according to different versions of SignalP software. (PDF)
