# Supplementary figures and images for: Evidence of Alternative Cystatin C Signal Sequence Cleavage Which Is Influenced by the A25T Polymorphism
Source: PLoS One. 2016 Feb 4;11(2):e0147684. doi: 10.1371/journal.pone.0147684 (PMC4741414; doi:10.1371/journal.pone.0147684)

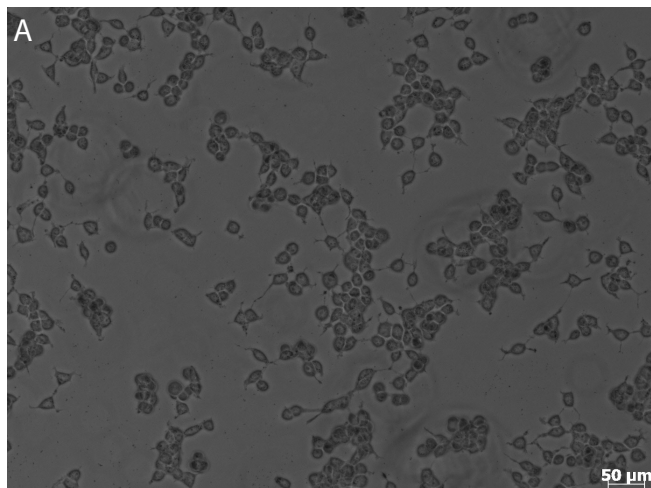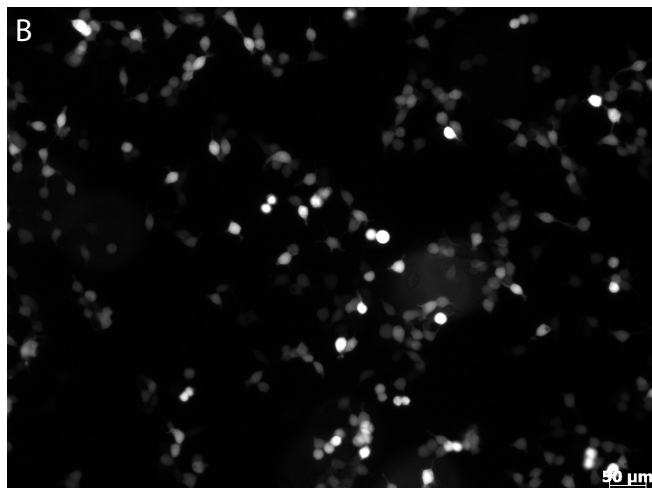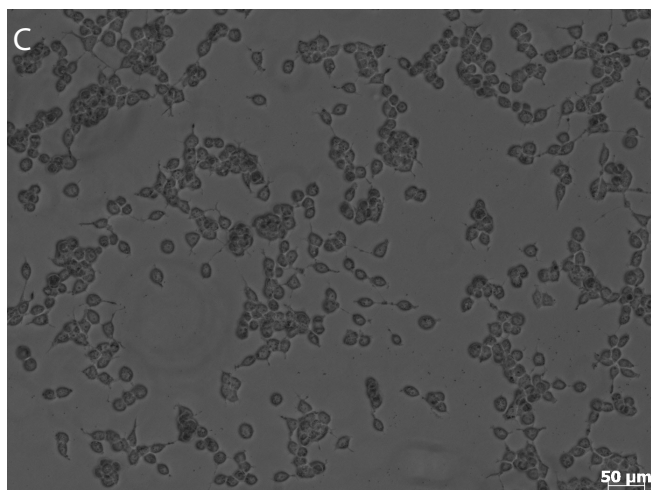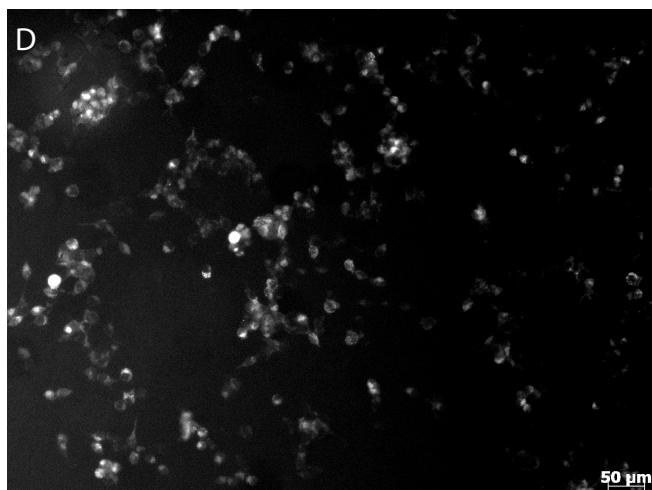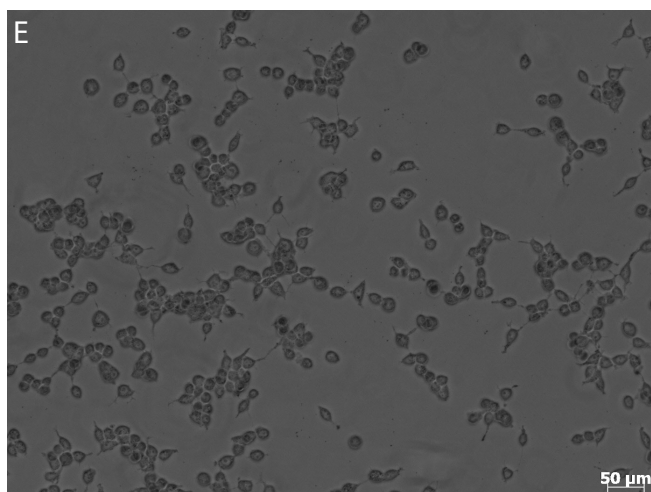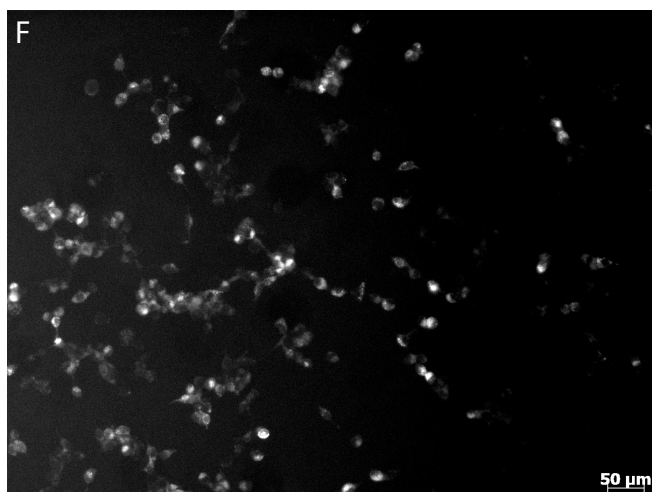

Supplement: S1 Fig — (A-F) HEK-293T cells were transfected with pEGFP-N1 (A, B), WT Cys C FLAG GFP (C, D), or A25T Cys C FLAG GFP (E, F) and imaged 24 h afterward. Bright field (A,C,E) and GFP-channel (B,D,F) images were captured. Transfection efficiencies for HEK-293T cells were consistently above 70%. (PDF) [file pone.0147684.s001.pdf]

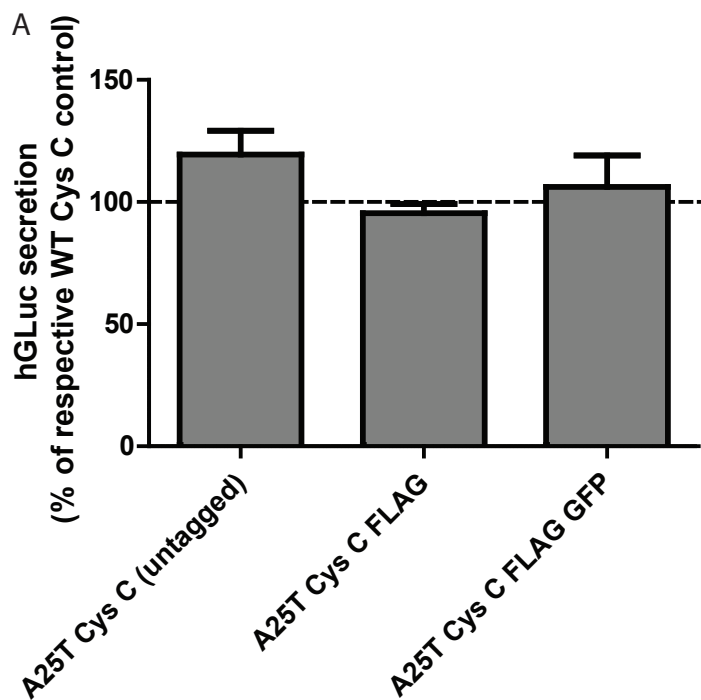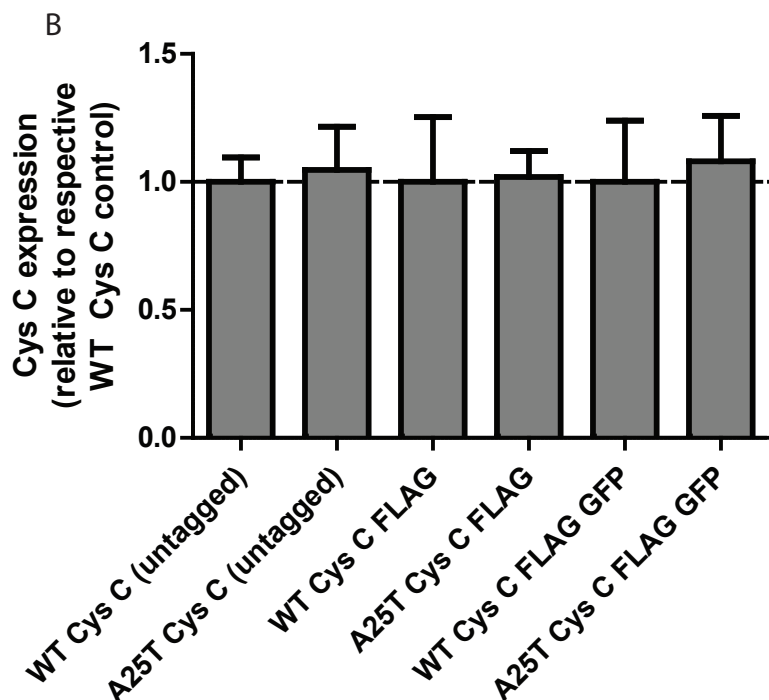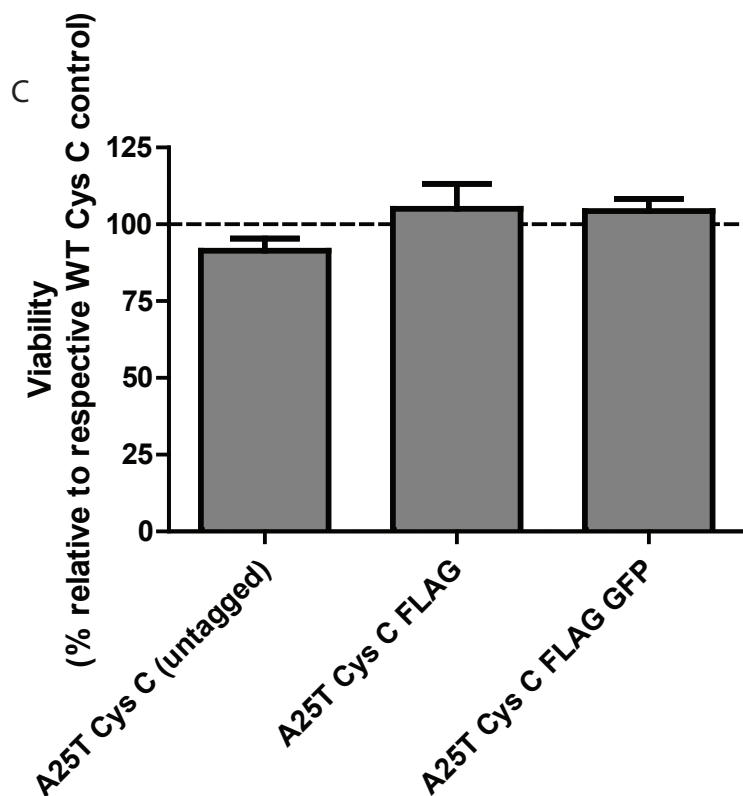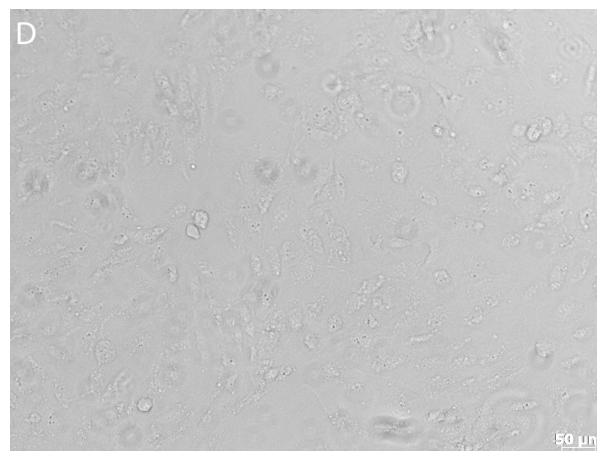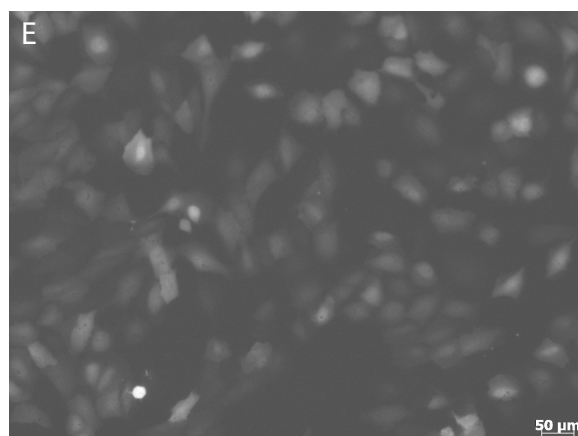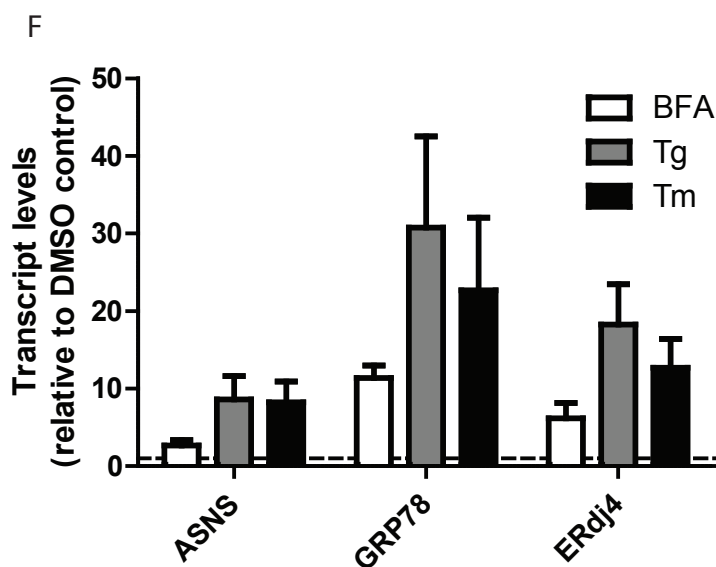

Supplement: S2 Fig — (A) Co-expression of a humanized, secreted Gaussia luciferase (hGLuc) construct along with Cys C demonstrates similar levels of another secreted protein after transfection. n = 3, mean ± S.D. (B) Cys C expression levels in HEK-293T cells 48 h after transfection. Representative data of at least three independent experiments. Mean ± 95% C.I. (C) Viability comparison of Cys C-expressing HEK-293T cells 48 h after transfection. n ≥ 3, mean ± S.D. (D, E) Representative transfection efficiency of ARPE-19 cells. ARPE-19 cells were transfected with pEGFP-N1 and imaged 24 h later using transmitted light (D) or a GFP filter set (E). Transfection efficiencies for ARPE-19 cells were consistently above 65%. (F) qPCR validation of UPR induction after BFA, Tg or Tm treatment of ARPE-19 cells. Representative data of three independent experiments, mean ± 95% C.I. (PDF) [file pone.0147684.s002.pdf]

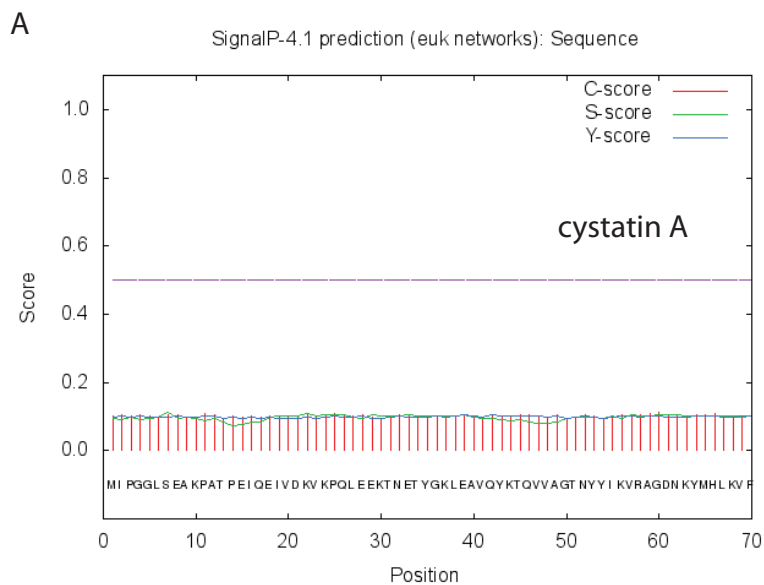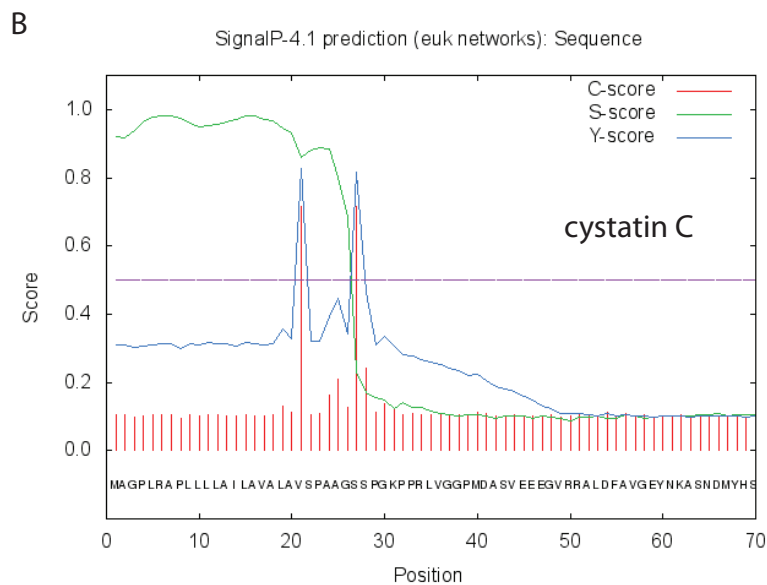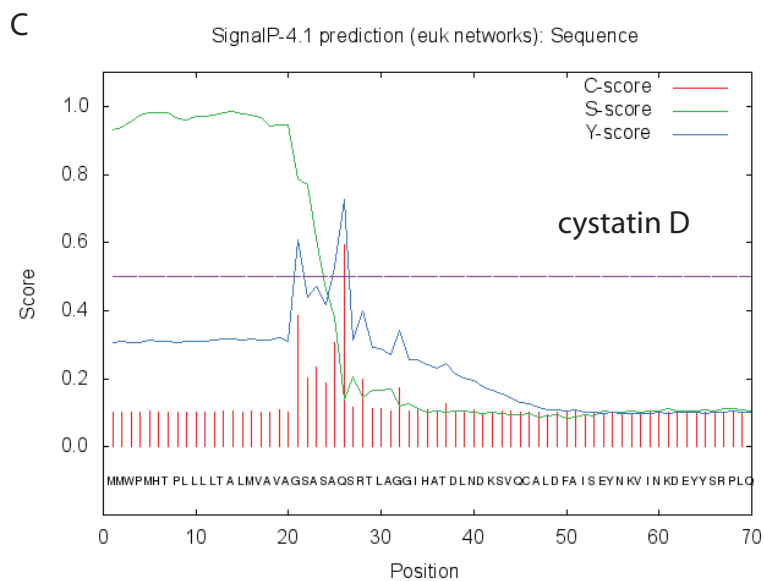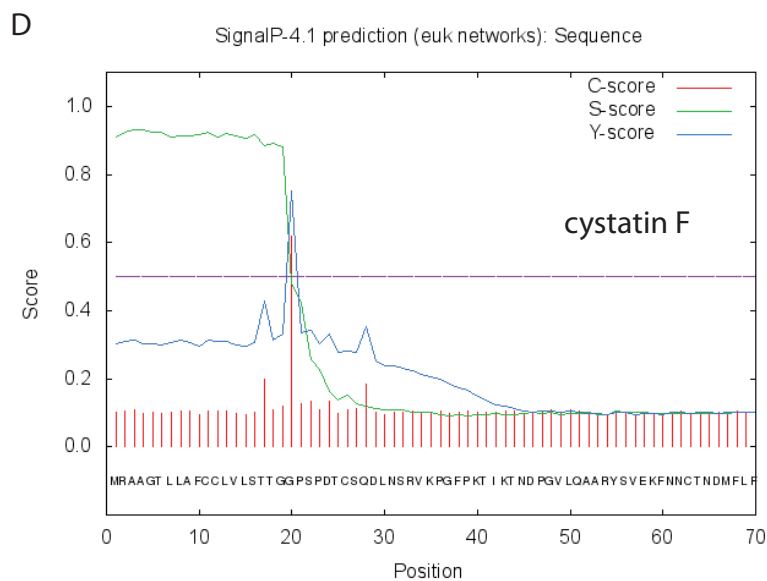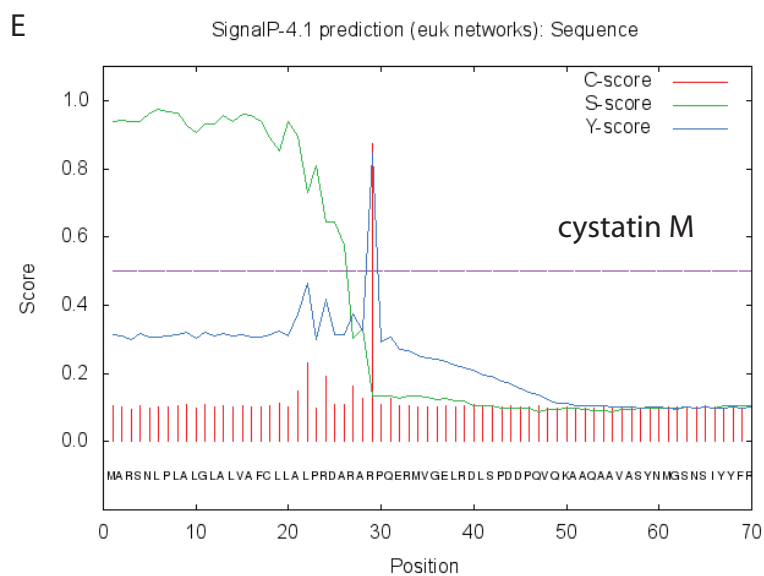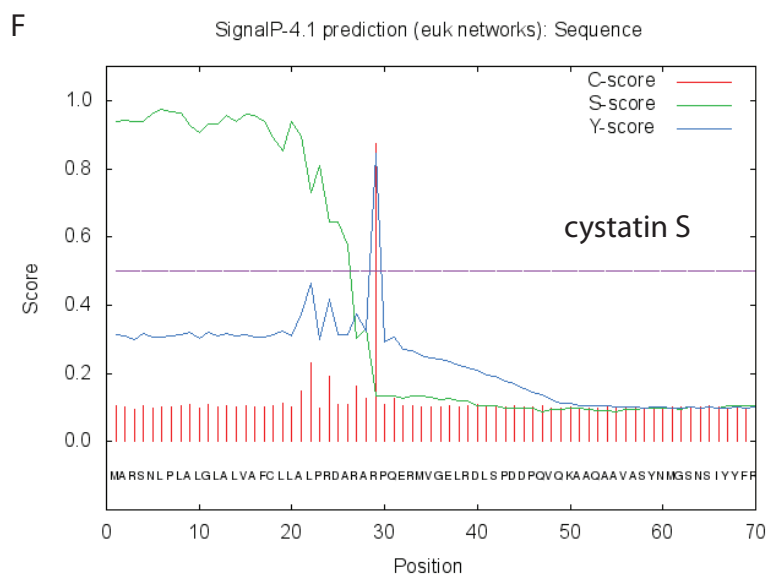

Supplement: S3 Fig — (A) SignaIP 4.1 prediction of cystatin A (a non-secreted cystatin), (B) cystatin C, (C) cystatin D, (D) cystatin F, (E) cystatin M and (F) cystatin S. (PDF) [file pone.0147684.s003.pdf]

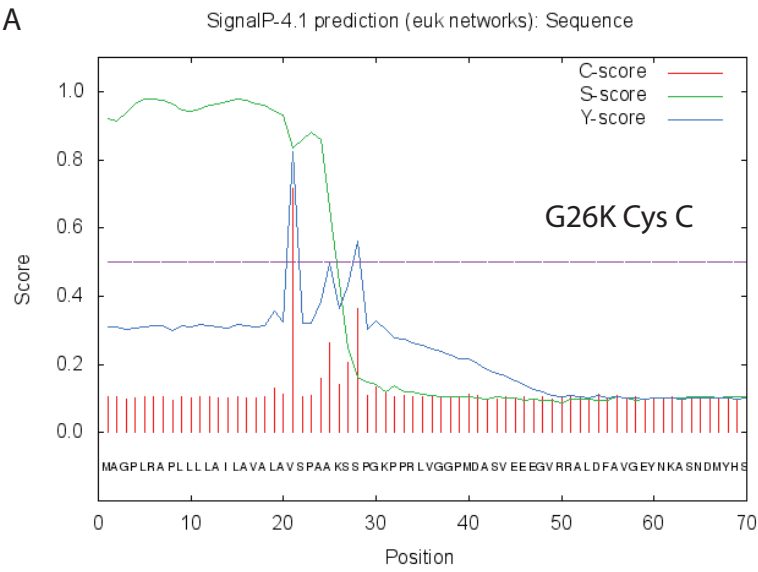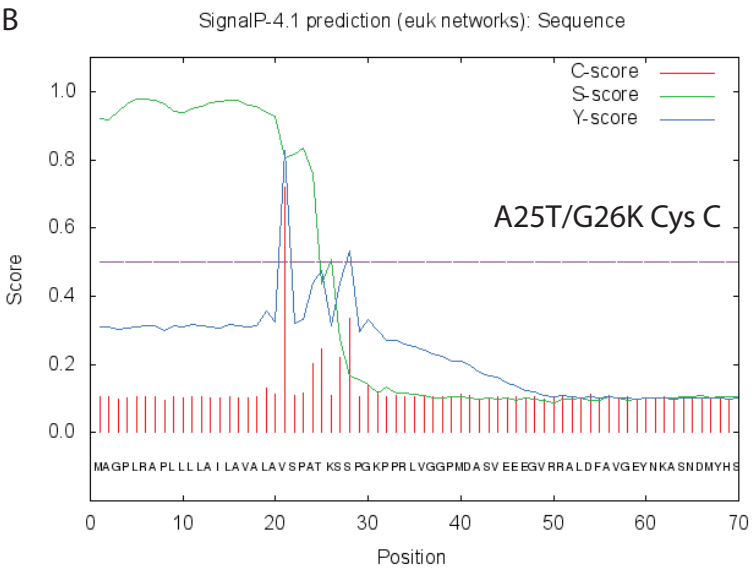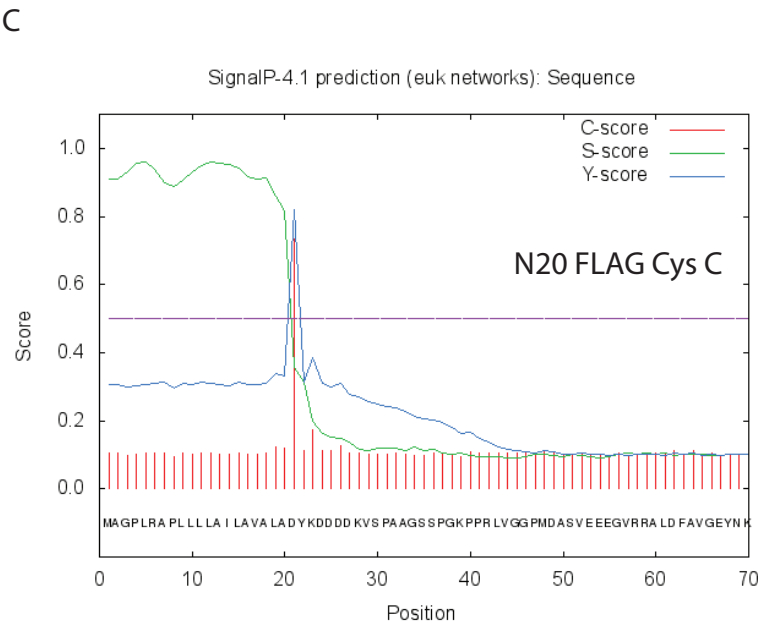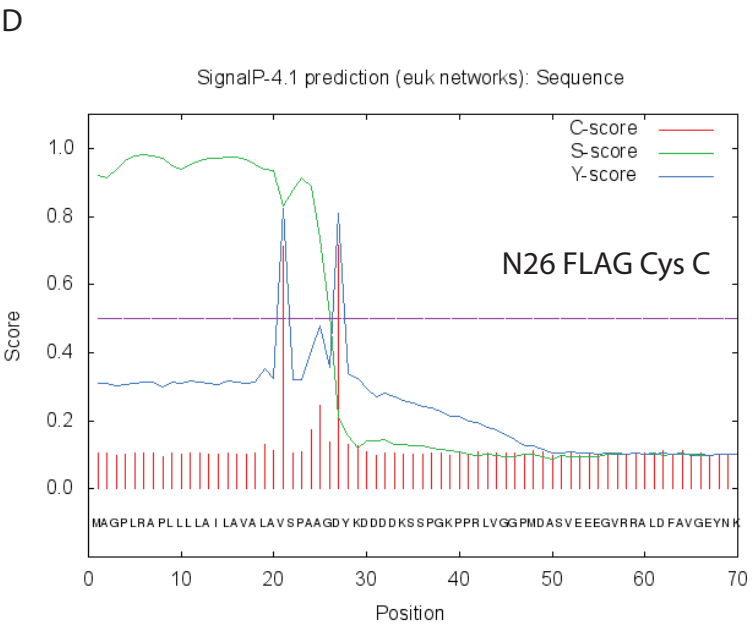

Supplement: S4 Fig — (A, B) SignaIP 4.1 prediction of G26K (A) or A25T/G26K Cys C (B). (C) SignaIP 4.1 prediction of N20 FLAG WT Cys C, which eliminates site 2 cleavage. (D) SignaIP 4.1 prediction of N26 FLAG WT Cys C, which maintains the two potential sites. Mutation of Ala25 to Thr did not change the predicted cleavage sites. (PDF) [file pone.0147684.s004.pdf]

A

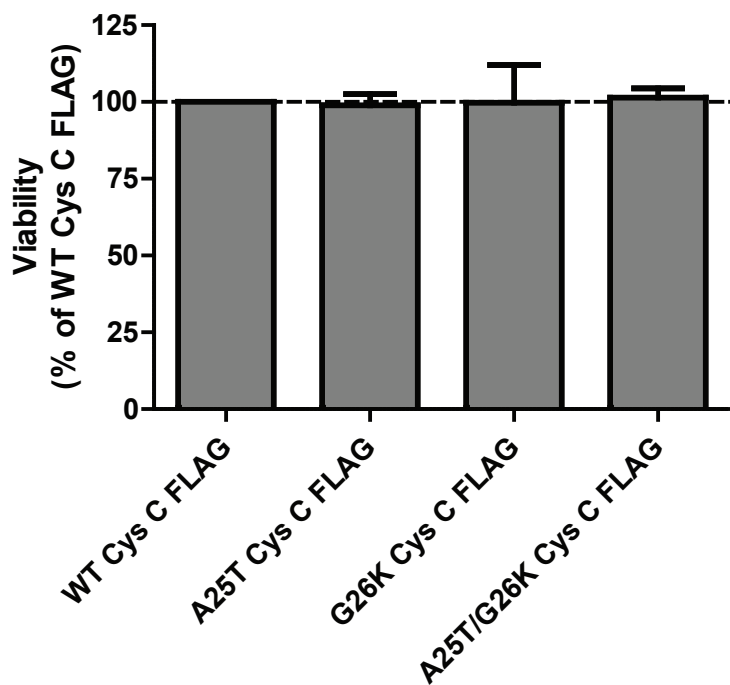

B

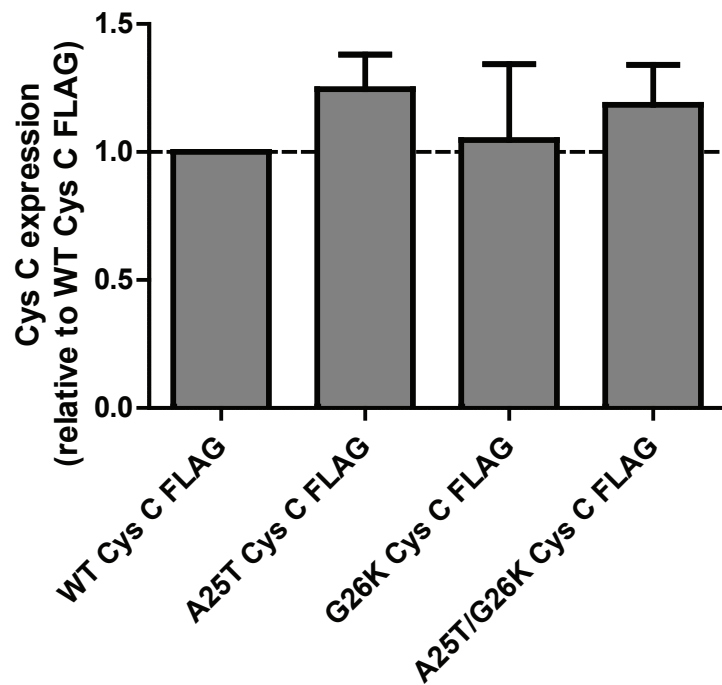

C

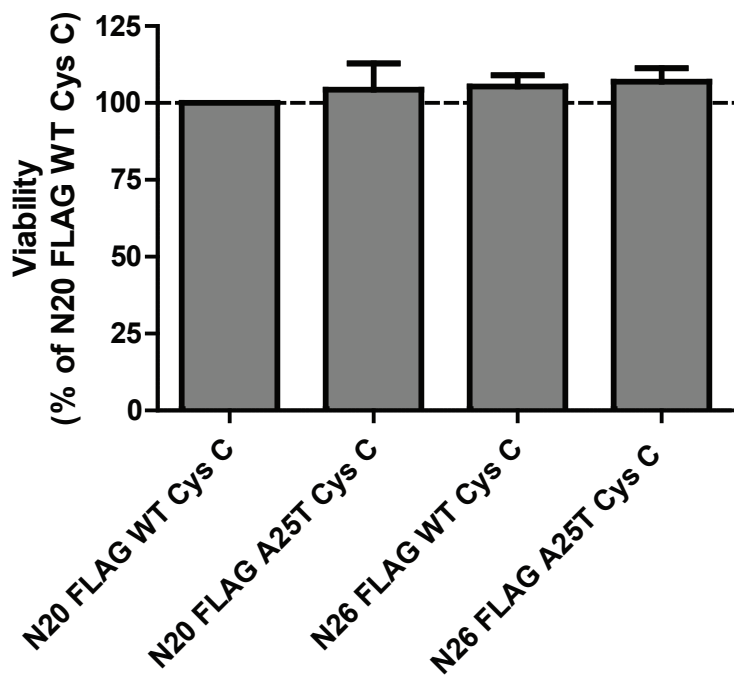

D

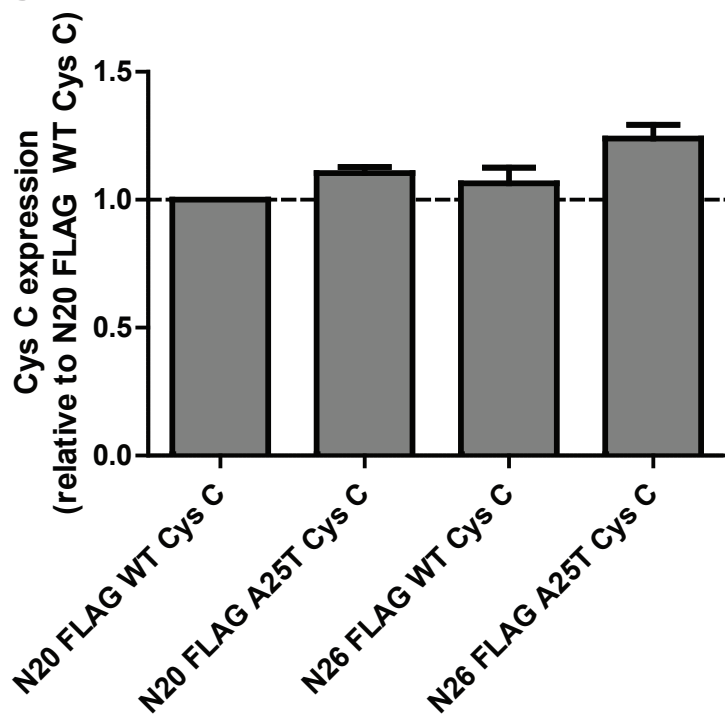

Supplement: S5 Fig — (A, C) Viability of the transfected HEK-293T cells was evaluated 48 h post transfection by the resazurin assay. (B, D) Cys C expression levels were monitored by qPCR 48 h after transfection. n ≥ 3, mean ± S.D. for all panels. (PDF) [file pone.0147684.s005.pdf]
