# Supplementary material for: Evidence of Alternative Cystatin C Signal Sequence Cleavage Which Is Influenced by the A25T Polymorphism
Source: PLoS One. 2016 Feb 4;11(2):e0147684. doi: 10.1371/journal.pone.0147684 (PMC4741414; doi:10.1371/journal.pone.0147684)
Supplement: S1 Table — (PDF) [file pone.0147684.s006.pdf]

version	predicted most likely signal seq cleavage site
SignalP 2.0	between Gly26 and Ser27
SignalP 3.0	between Gly26 and Ser27
SignalP 4.0	between Ala20 and Val21
SignalP 4.1	between Ala20 and Val21
